# Acute Kidney Injury in Pediatric Patients Treated with Vancomycin and Piperacillin-Tazobactam Versus Vancomycin and Cefotaxime: A Single-center Study

**DOI:** 10.7759/cureus.6805

**Published:** 2020-01-28

**Authors:** Rewaa Alqurashi, Mawaddah Batwa, Bashayer Alghamdi, Saja Aljohani, Nada Zaher, Amal Alzahrani, Eman Aldigs, Osama Safdar

**Affiliations:** 1 Internal Medicine, King Abdulaziz University, Jeddah, SAU; 2 Medical Microbiology and Parasitology, King Abdulaziz University, Jeddah, SAU; 3 Pediatrics, King Abdulaziz University, Jeddah, SAU

**Keywords:** infectious diseases, antibiotics, pediatrics, nephrology

## Abstract

Background

Previous literature showed a higher incidence of acute kidney injury (AKI) in pediatric patients using vancomycin + piperacillin-tazobactam compared to cefepime + vancomycin. Our aim was to compare the incidence of developing AKI during the use of vancomycin + cefotaxime with that during the use of vancomycin + piperacillin-tazobactam in pediatric patients.

Methods

This was a retrospective, matched cohort study that used electronic records from May 1, 2015 through April 30, 2018 for all patients aged less than 16 years who received intravenous (IV) vancomycin + piperacillin-tazobactam or cefotaxime + vancomycin for at least 72 hours. AKI was defined by Kidney Disease Improving Global Outcomes (KDIGO) guidelines. Each patient from the vancomycin + piperacillin-tazobactam group was matched 1:1 with those in the vancomycin + cefotaxime group according to their age, chronic disease, gender, and the number of concomitant nephrotoxic agents. A total of 64 cases were included. Statistical analysis was performed using descriptive statistics and binary logistic regression.

Results

AKI developed in 10 of 32 patients (31.25%) who were using vancomycin + piperacillin-tazobactam. On the other hand, 13 of 32 patients (40.62%) were using cefotaxime + vancomycin (p = 0.047). Of the 10 patients who were on vancomycin + piperacillin-tazobactam regimen, 80% developed AKI Stage I. Of the 13 patients who were using cefotaxime + vancomycin, 46% developed AKI Stage II, although no statistical significance was noted in all stages.

Conclusion

Our study showed that patients treated with cefotaxime and vancomycin showed a higher incidence of AKI than patients treated with vancomycin and piperacillin-tazobactam, although the study showed no statistical significance.

## Introduction

Drug exposure plays a significant role in the destruction of kidney function. Since kidneys are mainly responsible for maintaining homeostasis in the body and eliminating waste products, they are more susceptible to xenobiotics [1]. Acute kidney injury (AKI) is characterized by an abrupt decrease in kidney function within hours due to structural damage. AKI has worse outcomes in hospitalized patients [2]. Its definition has evolved from the RIFLE (Risk, Injury, Failure, Loss of Kidney Function, and End-stage Kidney Disease) criteria and the AKIN (Acute Kidney Injury Network) classification to the Kidney Disease Improving Global Outcomes (KDIGO) classification in 2012 [3-5]. KDIGO defines AKI on the basis of serum creatinine and urine output over a specific duration. Unlike the RIFLE criteria, which included glomerular filtration rate (GFR) as an important parameter, clinical judgment was required for patients to meet the AKI, RIFLE, and AKIN criteria, which were originally developed for average-sized adults and not for children [6].

Vancomycin (VAN) is a common nephrotoxic antibiotic, which has multiple FDA-approved and off-label clinical uses [7]. It was reported that 10% - 20% and 30% - 40% of patients had AKI caused by conventional and high doses of VAN therapy, respectively. The increased production of reactive oxygen species during oxidative stress was thought to be the most probable mechanism for its nephrotoxicity [7]. There are numerous documented risk factors that could potentiate the occurrence of VAN-induced nephrotoxicity. These risk factors include increased VAN levels (> 20 mg/L), administering high doses to patients (> 4 g/day), prolonged therapy (> 7 days), intensive care unit admittance (especially if it was a prolonged stay), and concomitant treatment with nephrotoxic agents, such as nonsteroidal anti-inflammatory drugs, loop diuretics, and more [2, 8].

Patients with suspected sepsis are at higher risk of developing drug-associated adverse effects due to the use of multiple antibiotic combinations (sepsis development in patients using VAN alone was 13.1% vs 27.2% in combination groups) [9-10]. In children under antibiotic treatment, the major adverse drug reactions were caused by β-lactams and VAN [11]. Piperacillin-tazobactam (PTZ) is an antipseudomonal antibiotic that inhibits β-lactam/β-lactamase and is used in combination with VAN for hospitalized patients [12]. Furthermore, cefotaxime is a third-generation cephalosporin that acts against gram-negative and some gram-positive bacteria. It is well distributed throughout the body and is excreted mainly unchanged in the urine [13]. Cefotaxime is used in combination with VAN to treat serious systemic infections (osteomyelitis, infective endocarditis, skin and soft tissue infection, and many others) [14]. 

Several studies have reported a higher incidence of AKI in adult patients using VAN + PTZ (VAN-PTZ) combination (16.3% to 34.8%) compared to VAN + cefepime, a fourth-generation cephalosporin (12.5% to 13.3%) [10, 15-17]. Similarly, higher rates of nephrotoxicity were observed among patients treated with VAN-PTZ compared with VAN treatment alone; however, the exact mechanism for the increased nephrotoxicity by this combination was not fully understood [18]. Moreover, a robust retrospective cohort study reported a higher incidence of AKI in pediatric patients using VAN-PTZ compared to VAN-cefepime [19].

However, there are no data regarding the incidence of AKI when cefotaxime is used in combination with VAN. Therefore, we tested the safety of VAN + cefotaxime compared to that of VAN-PTZ in a pediatric population. At the King Abdulaziz University Hospital, we prefer using cefotaxime over cefepime to avoid future cefepime resistance.

## Materials and methods

Study design

This study was approved by the Institutional Review Board (IRB) of the King Abdulaziz University Hospital (KAUH) Jeddah, Saudi Arabia. The data were collected from the pediatric system database, Phoenix Data Systems Asset Information Management System (Phoenix Data Systems, Southfield, MI), starting from May 1, 2015 to April 30, 2018. There were 1,129 medical file records of patients who had received IV VAN out of which we had to extract the data that matched our inclusion criteria.

Study subjects

Electronic records of all patients aged less than 16 years who received IV VAN-PTZ or IV VAN + cefotaxime for at least 72 hours were reviewed; baseline serum creatinine (measured within a week before antibiotic administration), plus daily creatinine levels during the antibiotic course were monitored. AKI was defined as an increase of serum creatinine (SCr) of > 0.3 mg/dl or an increase in SCr to ≥ 1.5 times baseline. If any patient met the criteria, he or she was defined to have AKI Stage 1, which was defined as a 1.5 to 1.9-fold increase in SCr from the baseline or SCr ≥ 0.3 mg/dL (≥ 26.5 µmol/L). Stage 2 was defined as a 2.0 to 2.9-fold increase in SCr from the baseline, while Stage 3 was defined as three-fold higher SCr or SCr ≥ 4.0 mg/dl (≥ 353.6 µmol/L).

Subject characteristics were also obtained from the records including age, gender, weight, height, chronic disease, indication for antibiotics, nephrotoxic agents, VAN trough (highest measurement that was taken in a steady-state prior to AKI development), therapy duration (at least one dose on a given day), and admittance to the intensive care unit (ICU).

Each patient from the VAN-PTZ group was matched 1:1 with those in the VAN-cefotaxime group according to their age (younger than three months, equal or between three months and one year, equal to or older than one year), chronic disease (congenital heart disease, malignancy, and others), gender, and the number of concomitant nephrotoxic agents.

Exclusion criteria

Patients with underlying kidney disease (preexisting renal dysfunction, structural kidney disease, undergoing dialysis, or AKI at admission) or did not have a match or who took oral VAN were excluded from the study (only patients who take IV antibiotics can be tracked properly in our hospital system). All patients who did not have a match in the other group were excluded from the study.

Statistical analysis

The collected data were entered using an online Google® drive form and transferred to Microsoft® Excel. Our statistical analysis was performed using the IBM® Statistical Package for Social Sciences (SPSS), v.23 software package (IBM SPSS Statistics, Armonk, NY). The incidence of developing AKI that was caused by VAN-PTZ or VAN + cefotaxime was calculated using bivariate logistic regression. Binary logistic regression was used for all variables (age, gender, weight, chronic disease, indication for antibiotics, nephrotoxic agents, VAN trough, therapy duration, and ICU admission) to determine their association with the incidence of developing AKI. We considered p-value < 0.05 as significant. All variables in each treatment group and AKI development were measured using descriptive statistics.

## Results

One-hundred and sixty-two patients were eligible for inclusion and 93 patients were excluded from the study because no match was found in the other treatment group. Five patients were from the Tazocin - vancomycin group and were excluded because there was no match for them in the other treatment group. A total of 64 cases were included: (n = 32 for VAN-PTZ) and (n = 32 for VAN + cefotaxime).

**Table 1:** Comparison of Clinical Characteristics in Matched Patients ^a^ Values shown are mean ± standard deviation or number + percentage. ^b^ Binary logistic regression (the association between variables and AKI) ARB: angiotensin II receptor blockers; CHD: congenital heart disease; CNS disease: central nervous system disease; GU/GI: genitourinary/gastrointestinal; ICU: intensive care unit; NSAIDS: nonsteroidal anti-inflammatory drugs; PTZ: piperacillin-tazobactam; SSTI: skin and soft tissue infection; VAN: vancomycin

Characteristics	VAN-PTZ (n = 32)^ a^	VAN-cefotaxime (n = 32)^ a^	P-value
Age (years)	1.33 (± 1.86)	1.29 (± 1.99)	0.492^b^
Male	18 (56.3%)	18 (56.3%)	0.126^ b^
Female	14 (43.8%)	14 (43.8%)	
Weight	8.28 (± 6.70)	7.96 (± 8.46)	0.619^ b^
Baseline serum creatinine (mg/dl)	0.3315 (± 0.17)	0.47 (± 0.29)	0.065^ b^
CHD	19 (59.4%)	19 (59.4%)	0.855^ b^
Malignancy	1 (3.1%)	1 (3.1%)	0.678^ b^
Hypertension	1 (3.1%)	4 (12.5%)	0.260^ b^
Hypotension	0 (0%)	1 (3.1%)	1.000^ b^
Primary immunodeficiency	1 (3.1%)	0 (0%)	1.000^ b^
Hypoalbuminemia	1 (3.1%)	0 (0%)	1.000^ b^
Others	0 (0%)	0 (0%)	1.000^ b^
Indication for antibiotics			
Empiric therapy	9 (28.1%)	6 (18.8%)	0.115^ b^
Prophylaxis	4 (12.5%)	7 (21.9%)	0.513^ b^
CNS disease	1 (3.1%)	1 (3.1%)	0.999^ b^
Bacteremia	3 (9.4%)	1 (3.1%)	0.641^ b^
Cardiovascular disease	7 (21.9%)	10 (31.3%)	0.600^b^
Respiratory disease	11 (34.4%)	8 (25%)	0.505^ b^
GU/GI disease	2 (6.3%)	2 (6.3%)	0.641^ b^
SSTI	1 (3.1%)	1 (3.1%)	0.678^ b^
Febrile neutropenia	1 (3.1%)	0 (0%)	1.000^ b^
Sepsis	12 (37.5%)	11 (34.4%)	0.141^ b^
Therapy duration (days)	8.47 (± 3.98)	6.50 (± 2.83)	0.600^ b^
Admittance to ICU	16 (50%)	15 (46.9%)	0.334^ b^
ICU duration (days)	5.69 (± 4.27)	3.53 (± 2.85)	0.970^ b^
Nephrotoxic medications			
Aminoglycosides	16 (50%)	8 (25%)	0.737^ b^
NSAIDS	6 (18.8%)	4 (12.5%)	0.265^ b^
Loop diuretic	20 (62.5%)	23 (71.9%)	0.802^ b^
Amphotericin B	2 (6.3%)	1 (3.1%)	0.923^ b^
Acyclovir	0 (0%)	1 (3.1%)	1.000^ b^
ARB	5 (15.6%)	6 (18.8%)	0.974^ b^
Contrast media	7 (21.9%)	3 (9.4%)	0.319^ b^
Vasopressor agents	6 (18.8%)	11 (34.4%)	0.949^ b^
Chemotherapeutic agents	1 (3.1%)	1 (3.1%)	0.999^ b^
Number of concomitant nephrotoxins	1.97 (± 1.09)	1.81 (± 1.06)	0.905^ b^
VAN trough	12.20 (± 4.84)	11.35 (± 4.79)	0.158^ b^

As seen in Table 1, the groups were well-balanced in terms of age, gender, weight, ICU duration, concomitant administration of nephrotoxic agents, chronic disease, sepsis, and baseline creatinine. 

Because sepsis contributes to the administration of multi-antibiotic combination, we included it in our matching criteria with a total number of 12 of 32 (37.5%) in the VAN-PTZ group and for the other group, the total numbers were 11 out of 32 (34.4%). The baseline creatinine in the VAN-cefotaxime group was higher than that in the VAN-PTZ group, although the association between the baseline creatinine and AKI was insignificant (P = 0.065).

AKI development in both treatment groups

AKI was noted in 10 out of 32 patients (31.25 %) who were using VAN+ PTZ and in 13 out of 32 patients (40.62%) who were using cefotaxime+ VAN, although there was no statistical significance between the two groups (p-value = 0.602) (Table 2). Most patients who were on VAN-PTZ regimen developed AKI Stage 1 (8/10, 80%), while most patients who were using cefotaxime + VAN developed AKI Stage 2 (6/13, 46%). No statistical significance was noted in all stages (Table 3).

**Table 2:** AKI Development in Both Treatment Groups AKI: acute kidney injury; PTZ: piperacillin-tazobactam; VAN: vancomycin

Characteristics		VAN-PTZ	VAN-cefotaxime
AKI	Yes	10 (31.25%)	13 (40.62%)
	No	22 (68.75%)	19 (59.37%)
Total		32	32

**Table 3:** AKI in Different Stages ^a  ^Values shown are numbers and percentages. AKI: acute kidney injury; PTZ: piperacillin-tazobactam; VAN: vancomycin

Characteristics	VAN-PTZ (n = 32)^ a^	VAN-cefotaxime (n = 32)^ a^	Total (n = 32)^ a^	P-value
Stage 1	8 (61.5%)	5 (38.4%)	13 (20.3%)	0.534
Stage 2	1 (3.1%)	6 (18.8%)	7 (10.9%)	0.109
Stage 3	1 (3.1%)	2 (6.3%)	3 (4.6%)	1.000

## Discussion

The purpose of our study was to detect the incidence of AKI during treatment with VAN-PTZ or VAN + cefotaxime combination in pediatric age groups. In this study, the hospitalized children who were exposed to VAN + cefotaxime were at a greater risk of AKI than were children exposed to the VAN-PTZ treatment combination (40.6% vs 31.3%). To our knowledge, this is the first matched cohort study showing a difference in the incidence of AKI among diverse pediatric populations treated with this antibiotic combination.

Previous studies investigated the nephrotoxicity of those two combinations by choosing one drug (cefepime) from the cephalosporin class as an example for the entire group [17, 20], but our results were inconsistent with theirs. Cook et al. evaluated the incidence of AKI development in VAN-PTZ vs VAN + cefepime in the pediatric population (VAN-PTZ 28.9% vs VAN + cefepime 7.9%, p-value = 0.001) [19]. Another unmatched study by Gomes et al. in 2014 showed that the VAN-PTZ combination caused AKI more frequently than the VAN + cefepime combination in adults (VAN-PTZ 34.8% vs VAN + cefepime 12.5%, p-value = 0.0001) [17].

Furthermore, a study by Al Yami on adults observed the relation between VAN with PTZ or with meropenem but did not reveal significant results between the two groups with respect to AKI development (VAN-PTZ 7.41% vs VAN + meropenem 5.33%, P = 0.4) [12].

In our study, AKI was noted in 31.3% of the subjects in the VAN-PTZ group vs. 40.6% in the VAN + cefotaxime group. The results were statistically insignificant (p-value = 0.602). Most patients who used the VAN-PTZ regimen developed Stage 1 AKI (8/10, 80%), whereas most VAN + cefotaxime patients developed Stage 2 AKI (6/13, 46%); again, no statistical significance was noticed in all AKI stages. This shows that VAN + cefotaxime is as nephrotoxic as VAN-PTZ; therefore, the regimen should be used with caution, especially in children with underlying kidney disease. However, the mechanism of its nephrotoxicity is not fully understood. Additional studies are needed to validate this hypothesis. Resolution or long-term sequelae from AKI are uncertain. Data were not collected.

This study has several limitations. First, some data were difficult to obtain from the database and were taken manually (therapy duration and baseline and daily serum creatinine). Some data were missing (adjusted weighted VAN). The second issue was that this is a single-center study.

## Conclusions

In conclusion, our study showed that cefotaxime + VAN is as nephrotoxic as VAN-PTZ. The mechanism is not understood. Since our study is the first to focus on cefotaxime + VAN, we believe that a multicentric study is necessary to confirm our results. After confirmation, we recommend decreasing the use of the VAN + cefotaxime regimen, especially in neonates, considering their immature kidneys.
